# The prognostic model based on tumor-associated neutrophils contributes to the stromal landscape and influences metabolic reprogramming in colorectal cancer

**DOI:** 10.3389/fimmu.2025.1587947

**Published:** 2025-09-02

**Authors:** Xinke Yin, Yuanling Jiang, Jinghua Huang, Jiahuan Luo, Liang Xu, Qiong Yang

**Affiliations:** ^1^ Department of Pathology, Sun Yat-Sen Memorial Hospital, Sun Yat-Sen University, Guangzhou, China; ^2^ Cellular & Molecular Diagnostics Center, Sun Yat-Sen Memorial Hospital, Sun Yat-Sen University, Guangzhou, China; ^3^ Department of Pathology, The Sixth Affiliated Hospital, Sun Yat-Sen University, Guangzhou, China; ^4^ Department of Oncology, Sun Yat-Sen Memorial Hospital, Sun Yat-Sen University, Guangzhou, China

**Keywords:** colorectal cancer, single cell sequencing, tumor-associated neutrophils, prognostic stratification, immune microenvironment, metabolic reprogramming

## Abstract

**Background:**

The tumor microenvironment (TME) is highly complex and significantly influences cancer prognosis and drug sensitivity. Tumor-associated neutrophils (TANs) play a key role in the TME. In this study, we aimed to investigate the TANs-related markers in colorectal cancer (CRC) and develop an integrated signature for prognostic stratification.

**Methods:**

The CRC single-cell RNA sequencing (scRNA-seq) data and RNA-seq data were obtained from TCGA and GEO. A risk score was calculated based on the 18 TAN-associated genes identified in CRC by scRNA-seq data and LASSO regression. Prognosis, stromal and immune infiltration landscape, metabolism, and treatment response were then investigated in the low- and high-risk score clusters using RNA-seq data.

**Results:**

Patients with a High-risk score had a significantly worse survival outcome than those with a Low-risk score (p < 0.0001). The prognostic predictive potency of the risk score was validated in both the TCGA validation cohort (p < 0.0001) and the GEO cohort (p < 0.00015). The areas under the curves of 1-, 3-, and 5-year survival were 0.76, 0.74, and 0.70 in the TCGA training set; 0.78, 0.68, and 0.78 in the TCGA validation set; and 0.65, 0.64, and 0.62 in the GEO set. The risk score was related to T, N, and M stages. A prognostic nomogram was constructed, and the predictive accuracy was assessed by calibration curve analysis. Decision curve analysis showed the clinical utility of the nomogram. Furthermore, the High-risk score cluster was significantly associated with the levels of cancer-associated fibroblasts, as well as activity in the transforming growth factor-β and WNT pathway. In depth, the High-risk score cluster exhibited lower levels of amino acid, tricarboxylic acid, and nucleotide metabolism, as well as poorer responses to chemotherapeutic agents such as 5-fluorouracil.

**Conclusion:**

This novel TAN subtype, based on 18 prognostic-related genes, could provide new insights into the prognostic stratification and treatment options for CRC.

## Introduction

Colorectal cancer (CRC) is the third most common cancer. Despite the availability of various treatments, it remains the second leading cause of cancer-related death worldwide (1). In recent years, the tumor microenvironment (TME) has emerged as a promising therapeutic target due to its potential to reduce the risk of distant metastasis, tumor recurrence, and treatment resistance, while improving therapeutic efficacy (2). However, targeting the pro-tumorigenic TME presents considerable challenges, as it exhibits a dual role in tumorigenesis, capable of exerting both beneficial and detrimental effects (3). The TME is a complex network around the tumor cells including fibroblasts, endothelial cells, the extracellular matrix, and immune cells (4). Therefore, distinguishing the contradictory components of the TME and targeting the desired subsets could offer novel strategies to disrupt the pro-tumorigenic microenvironment and improve therapeutic outcomes.

Neutrophils, as a part of the TME, have traditionally been regarded as inert bystanders. However, emerging evidence indicates that neutrophils exhibit plasticity affected by the TME and can be divided into four types, including N1/N2 neutrophils, polymorphonuclear neutrophil myeloid-derived suppressor cells, and tumor-associated neutrophils (TANs), which play significant roles in either opposing or promoting cancer progression (5). Meanwhile, TANs have an indirect impact on the TME and its immune composition, making them a promising area to explore (6). The capacity to clinically target TANs, either by suppressing or activating them, could make a significant achievement in understanding their role in cancer progression and accelerate the emergence of novel therapies. However, defining neutrophil populations remains challenging, as many subsets lack specific cell surface markers and are primarily characterized by their functional phenotypes (7). To date, studies on the relationship between TANs and survival have focused on TAN levels (8), the ratio of infiltrating TANs to plasma cells (9), and the specific location of TANs (10). Given the general consensus that neutrophils can express various cell surface markers and receptors, many of which are clinically relevant. Therefore, exploring multiple markers associated with CRC prognosis and treatment, in addition to generic single neutrophil markers (e.g. CD66b), could reveal novel neutrophil subtypes in the CRC.

In recent years, single-cell RNA sequencing (scRNA-seq) has enabled the precise differentiation of cell origins and the inference of population dynamics (11). It has become a crucial tool for identifying potential biomarkers, particularly for TANs with high plasticity. In this study, we analyzed 133 TANs cluster marker genes using scRNA-seq data. Then, a novel TAN subtype was explored based on 18 prognostic-related genes that were identified by LASSO regression analysis. Their regression coefficients were determined by multivariate Cox regression, and patients was stratified into two clusters according to the risk score. The risk score proved to be an excellent biomarker for predicting prognosis. Subsequently, a nomogram model integrating the risk score and clinicopathologic characteristics was established, demonstrating significant clinical utility. Finally, we investigated the response of the two clusters to extracellular matrix-related processes, immunotherapy, and chemotherapy.

## Materials and methods

### Public datasets

**Figure 1:**
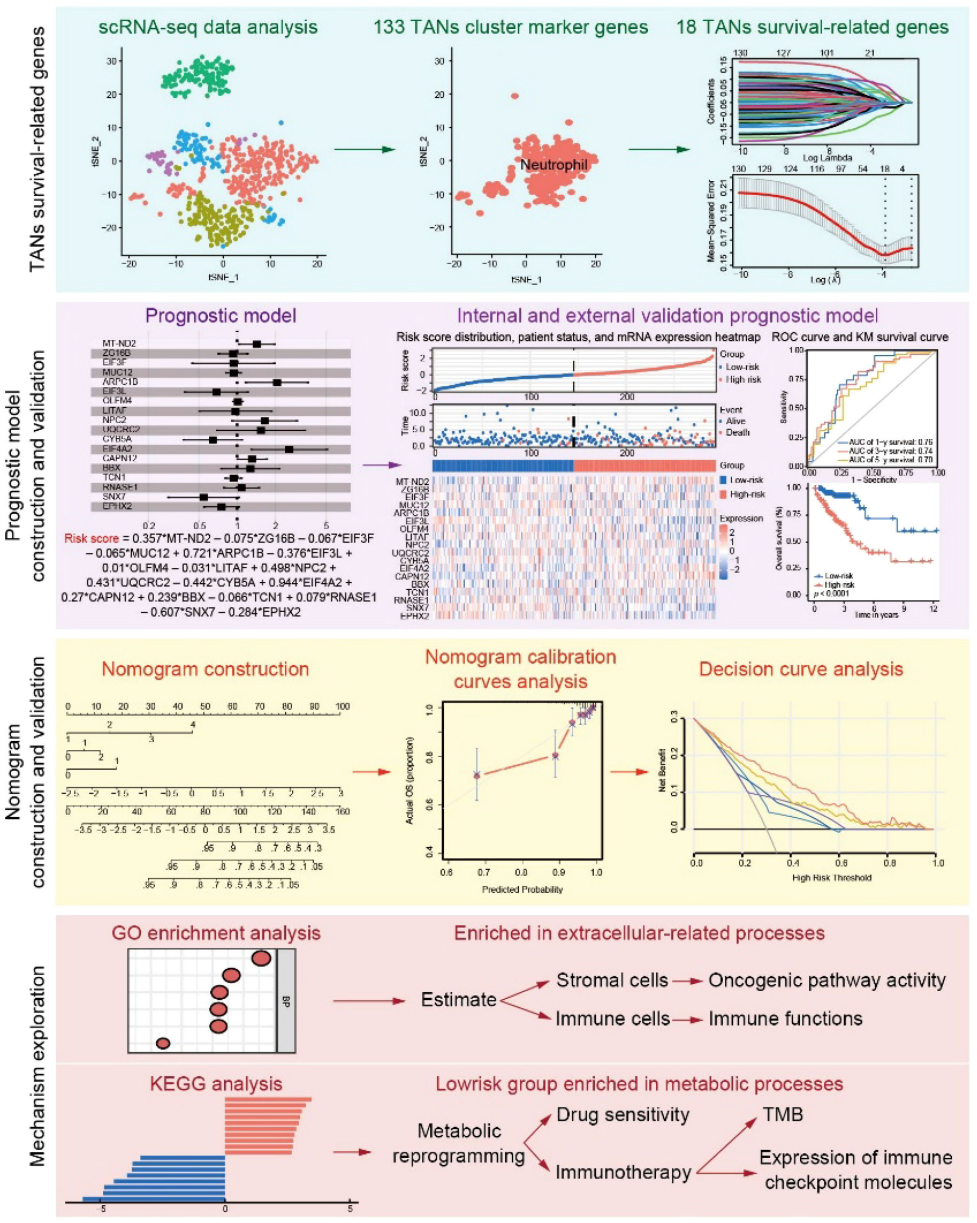
Workflow of the study. scRNA-seq data were obtained from the GEO and were used for TANs-related marker gene selection. After LASSO regression analysis, the 18 TANs survival-related genes were generated and further used to calculate the risk score. The performance of the risk score in predicting prognosis was validated in the internal and external cohorts. A nomogram combining clinical characteristics and the risk score was constructed and validated. GO and KEGG analyses were further used to explore the mechanism and treatment response prediction.

The scRNA-seq data (GSE163974) were obtained from the Gene Expression Omnibus (GEO) database, available at https://www.ncbi.nlm.nih.gov/geo/. This dataset included three cancer tissues (GSM4994386) and three normal tissues (GSM4994385), with GSM4994386 used for analysis in this study. Bulk CRC RNA-seq data were retrieved from the GEO database (referred to as the GEO cohort), including GSE17536, GSE39084, and GSE39582, as well as from the UCSC Xena GDC Hub (https://xenabrowser.net/datapages/, referred to as the TCGA cohort). The TCGA cohort comprised 496 CRC patients with complete gene expression and clinical data. Batch effects in the GEO cohort were corrected using the ‘combat’ algorithm implemented in the R package ‘sva’ (v3.50.0), and 818 CRC patients with sufficient gene expression and survival data were included in this study. The analysis workflow for all datasets is shown in the flowchart in **Figure 1**.

### Comprehensive scRNA-seq data analysis and cell cluster annotation

The R package ‘Seurat’ (v4.4.0) was used to analyze the scRNA-seq data. First, cells that failed quality control were excluded from the dataset based on the following criteria (**Supplementary Figure S1A**): 1) 200 < nFeature_RNA < 4000; 2) percent.mt < 20. The analysis was performed following the standard workflow of Seurat (12) and the top 20 principal components (PCs) were used to analyze the cell subset according to the JackStraw algorithm (**Supplementary Figures S1B, C**). Marker genes of the identified clusters were found using the FindAllMarkers function (min.pct = 0.25, logfc.threshold = 0.25). PanglaoDB (https://panglaodb.se/search.html) and CellMarker (http://117.50.127.228/CellMarker/) were then used to annotate cell subsets. Finally, 133 cluster marker genes of the TANs clusters were obtained (**Supplementary Table S1**).

### Calculation and validation of the TANs-based prognostic risk model

For the construction of a prognostic risk model, the TCGA cohort was divided into a training set (n = 291) and an internal validation set (n = 290) using the R package ‘caret’ (v6.0-94), and the GEO cohort (n = 818) was applied as an external validation set. To identify reliable prognosis-related genes, LASSO regression was performed in the training cohort. The size of the penalty term of the LASSO regression was controlled by λ, which selected from the R package ‘glmnet’ (v4.1-8). The regression coefficients of the selected genes were then obtained from multivariate Cox regression. The R packages ‘survminer’ (v0.4.9) and ‘survival’ (v3.5-7) were used to perform forest plots, Cox PH model assumptions, Kaplan-Meier survival curves, and time-dependent receiver operating characteristic (ROC) curves.

### Construction and validation of a prognostic nomogram based on TANs

To predict the prognosis of CRC patients, a nomogram combining the TANs-related genes and clinical characteristics was constructed using the R package ‘rms’ (v6.7-1). The relationship between risk clusters and TNM stage was explored using the R package ‘ggstatsplot’ (v0.12.1). Time-dependent calibration curves were used to evaluate the nomogram model, and 1000 boot-strapping resamples were performed for internal and external validation. To assess the clinical utility of the prognostic nomogram, decision curve analysis was performed using the R package ‘rmda’ (v.1.6).

### Enrichment analysis

The differentially expressed genes (DEGs) (|lgFC| ≥ 0 and p value ≤ 0.05) between the two groups (Low-risk vs. High-risk) were obtained from the TCGA cohort using the R package ‘limma’ (v3.58.1). Kyoto Encyclopedia of Genes and Genomes (KEGG) and Gene Ontology (GO) were conducted using the R package ‘clusterProfiler’ (v4.10.0) based on the DEGs. Visual maps with annotations were illustrated using the R package ‘enrichplot’ (v.1.22.0).

### Immune infiltration landscape and metabolic reprogramming

Firstly, the R package GSVA (v1.40.1) was used to perform single-sample gene set enrichment analysis (ssGSEA) for each sample. Scores including Stromal, Immune and ESIMATE were then calculated using the R package ‘estimate’ (v1.0.13). Further, the scores of endothelial cells and fibroblasts were evaluated using the ‘MCPcounter’ and ‘EPIC’ methods in the R package ‘IOBR’ (v0.99.8). In addition, 28 immune cells, 13 immune functions (13) were evaluated using ssGSEA, and 7 metabolic signatures (14). Expression levels of immune-related molecule in Low-risk and High-risk clusters was displayed using box plots.

### Somatic variants and tumor mutation burden score

Colorectal adenocarcinoma variant data were obtained directly from the TCGA database using the R package ‘TCGAmutations’ (v0.3.0). The R package ‘maftools’ (v2.18.0) was performed to summarize, analyze and visualize the Mutation Annotation Format files and generate the OncoPrint plot of TANs-related genes. In addition, the TMB score was calculated using the ‘tmb’ algorithm.

### Oncogenic pathway activity and treatment prediction

Activation of cell signaling pathways between the Low-risk and High-risk clusters was examined using the R package PROGENy (v1.24.0). To explore the response of chemotherapeutics and inhibitors, the concentration that causes a 50% reduction in growth (IC50) of chemotherapeutics and inhibitors was calculated using the R package ‘pRRophetic’ (v0.5).

### Statistical analysis

All statistical analyses were performed using R version 4.3.2. Kaplan-Meier survival curves were generated and compared using the log-rank test. Statistical comparisons between groups were performed using non-parametric tests. Statistically significant was defined as *p* < 0.05.

## Results

### scRNA-seq analysis and identification of the TANs cluster in CRC

**Figure 2:**
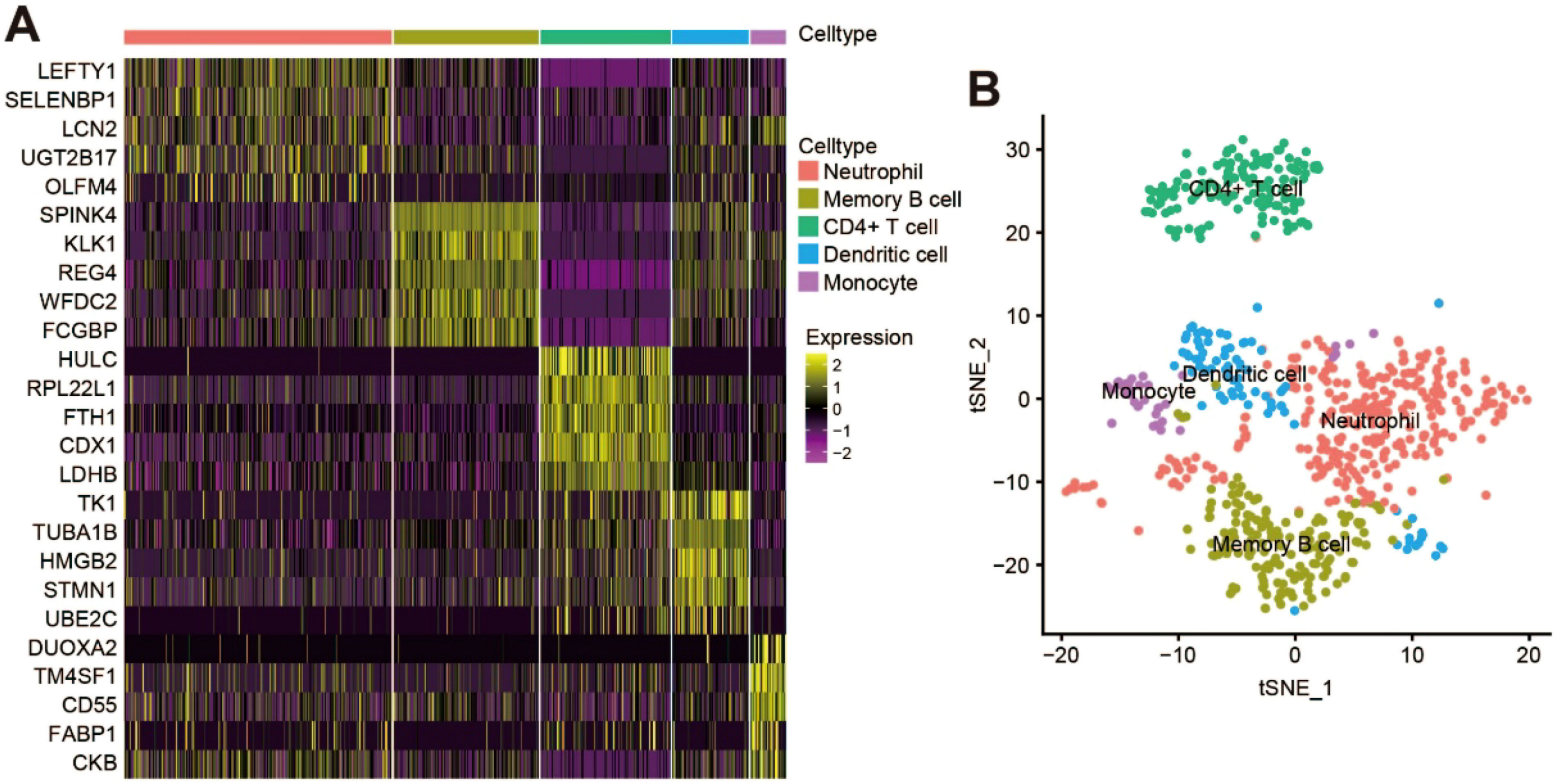
scRNA-seq analysis and identification of TANs cluster in CRC. **(A)** Heatmap showing the top five cluster marker genes of each cell type; **(B)** t‐Distributed stochastic neighbor embedding (tSNE) visualization of the scRNA cell clusters and annotation.

All genes from CRC samples in GSE163974 were standardized and corrected, and 2,000 variable genes were identified (**Supplementary Figure S1D**). After principal component analysis (PCA) dimensionality reduction analysis, cluster marker genes were screened and the top 5 genes with dominant contribution in each cluster were presented (**Figure 2A**). Furthermore, five cell types (CD4+ T cells, monocytes, dendritic cells, neutrophils, and memory B cells) were defined after manual annotation and visualized by t-distributed stochastic neighbor embedding (**Figure 2B**). Finally, 133 cluster marker genes (**Supplementary Table S1**) were extracted from the TANs cluster.

### Establish a stable and effective TANs-based prognostic risk model

**Figure 3:**
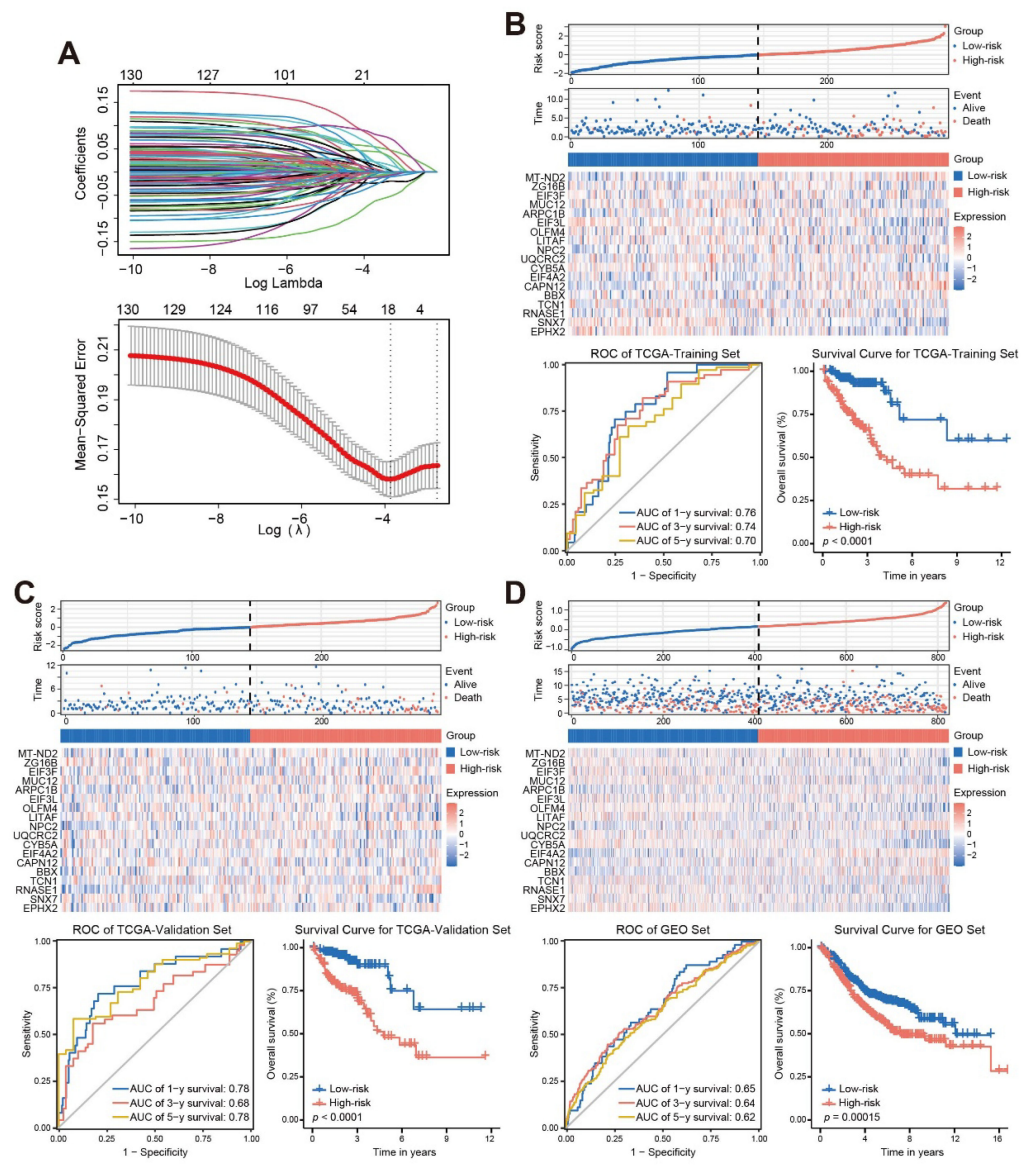
Establish a stable and effective TANs-based prognostic risk model. **(A)** Eighteen TANs-related genes with prognostic characteristics were screened by the LASSO regression algorithm; **(B-D)** The risk score distribution, patient status, mRNA expression heatmap, ROC curve, and survival curve of the training sets **(B)**, internal validation set **(C)**, and external validation set **(D)**. ROC, receiver operating characteristic; AUC, area under the curve.

LASSO regression analysis (**Figure 3A**) identified 18 genes (shown in **Supplementary Table S1**) with prognostic value, which were mainly expressed in the neutrophil cluster (**Supplementary Figure S2A**). Multivariate Cox regression was then performed to obtain the regression coefficients of the 18 genes (**Supplementary Figure S2B**), and the final prognostic model was as follows: Risk score = 0.357*MT-ND2 – 0.075*ZG16B – 0.067*EIF3F –0.065*MUC12 + 0.721*ARPC1B – 0.376*EIF3L + 0.01*OLFM4 – 0.031*LITAF +0.498*NPC2 + 0.431*UQCRC2 – 0.442*CYB5A + 0.944*EIF4A2 + 0.27*CAPN12 + 0.239*BBX – 0.066*TCN1 + 0.079*RNASE1 – 0.607*SNX7 – 0.284*EPHX2.

Kaplan-Meier survival curves and time-dependent ROC curves were constructed to evaluate the prognostic performance of the risk score. The results of the Kaplan-Meier survival curve analysis showed that patients with a High-risk score had a worse outcome than that those with a Low-risk score (log-rank, *p* < 0.001). The area under the curve (AUC) values for 1-year, 3-year, and 5-year in the TCGA-training set, TCGA-validation set and GEO set were all greater than 0.6. These results demonstrated that the risk score could be used as a predictor of patient prognosis (**Figures 3B-D**).

### Clinical application of the prognostic risk model

**Figure 4:**
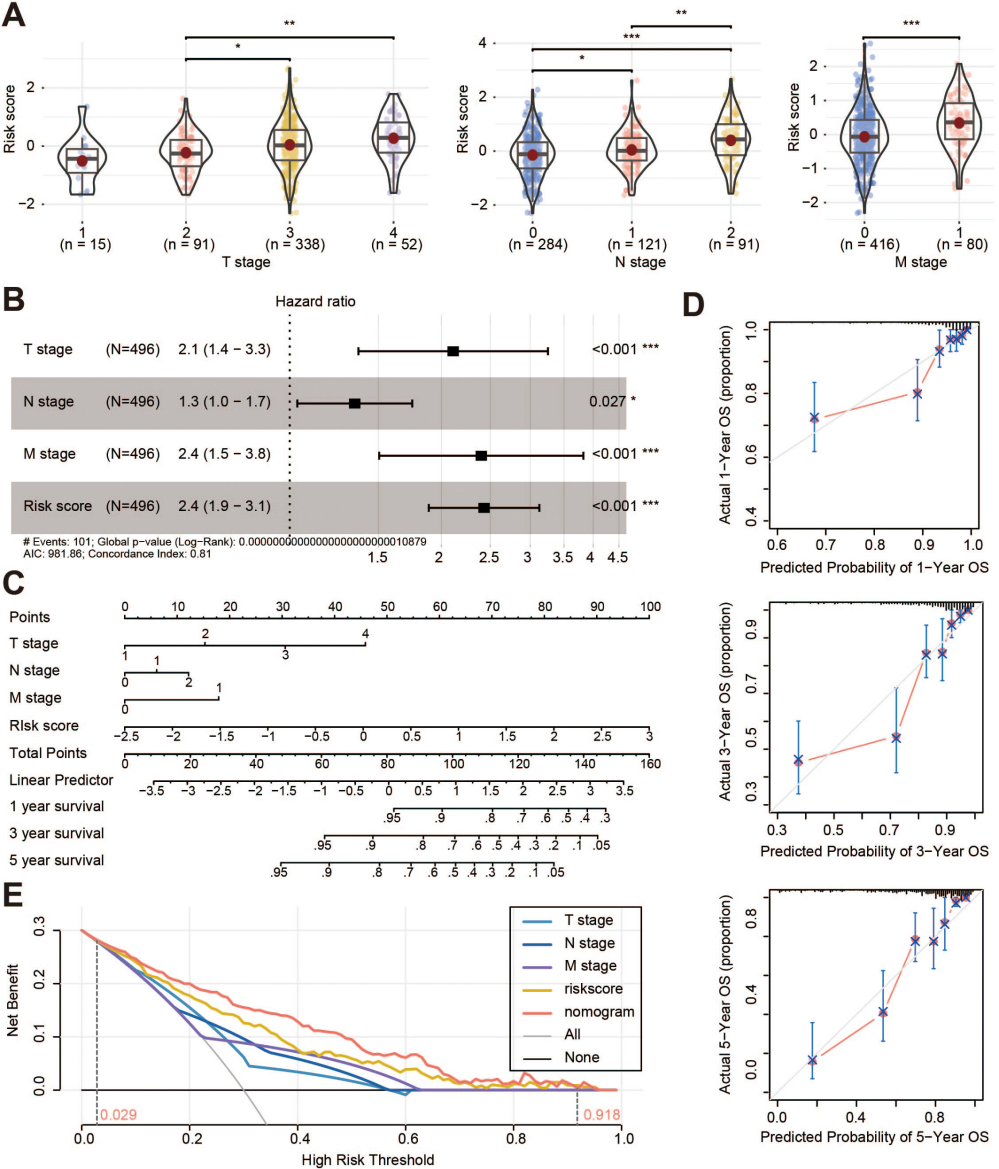
Clinical application of the prognostic risk model. **(A)** Correlation analysis between risk score and T stage, N stage, and M stage of the TCGA cohorts; **(B)** Multivariate cox regression analysis of the clinical information and risk score; **(C)** Nomogram of the TANs-related prognostic risk model; **(D)** Calibration curves of the nomogram for predicting 1-, 3-, and 5-year survival; **(E)** Decision curve analysis for the prognostic risk nomogram. OS, overall survival. *p < 0.05; **p < 0.01; ***p < 0.001.

The comparison of clinicopathological features with the risk score was explored, and the results showed that T, N and M stages were significantly associated with the risk score (**Figure 4A**). Multivariate analysis showed that T stage, N stage, M stage, and risk score were the independent prognostic factors of OS (all *p* < 0.05, **Figure 4B**; **Supplementary Figure S3A**). These characteristics were then integrated into a prognostic nomogram (**Figure 4C**). For both the TCGA cohort (**Figure 4D**) and the GSE39582 cohort (**Supplementary Figure S3B**), the calibration curves for 1-year, 3-year, and 5-year survival showed that the nomogram predicted survival rates closely matched the actual survival rates. Decision curve analysis (DCA) showed that the nomogram provided greater benefit than clinicopathological features alone or risk score alone when a patient’s threshold probability ranged from 2.9% to 91.8% (**Figure 4E**).

### Tumor microenvironment in the high-risk cluster

**Figure 5:**
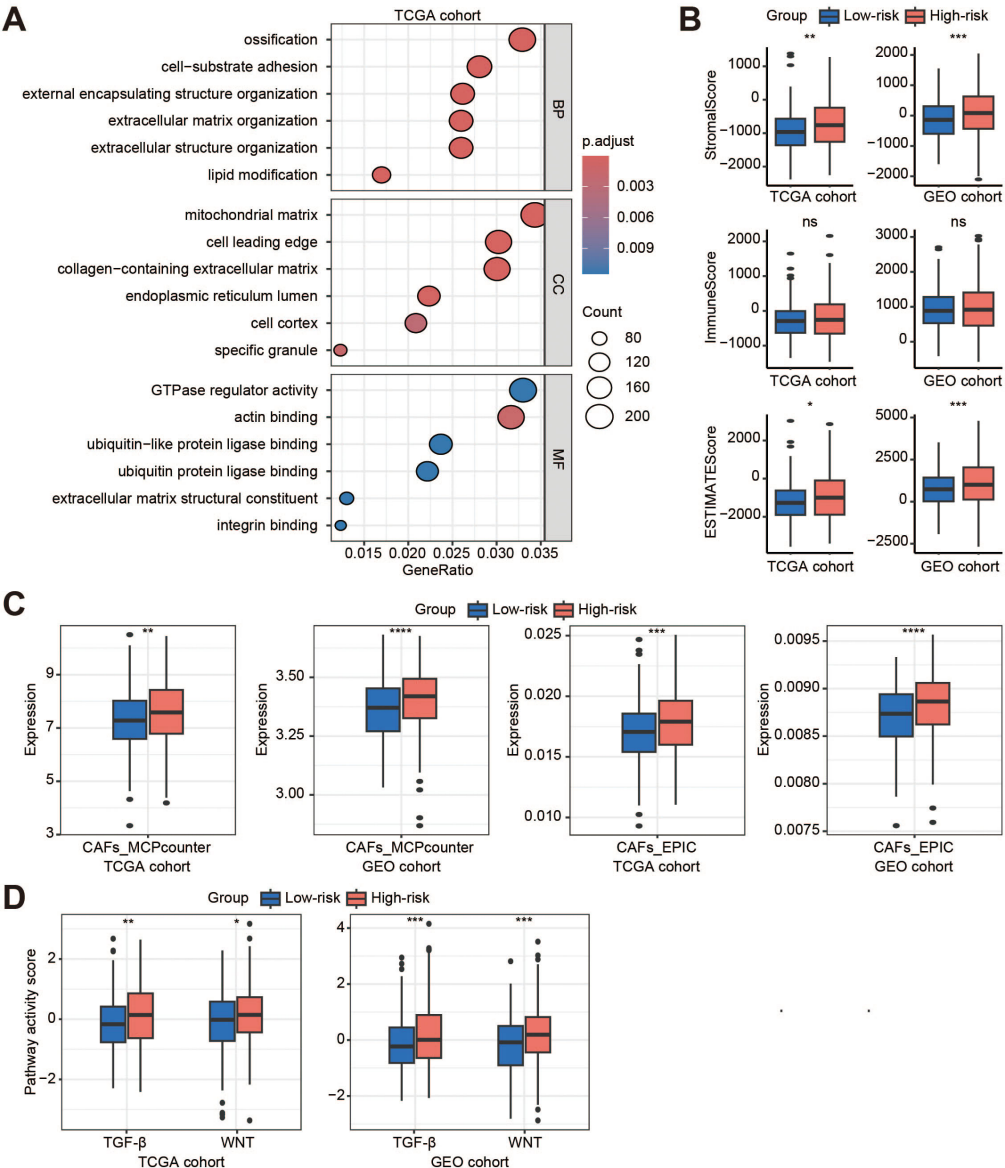
Tumor microenvironment in the high-risk cluster. **(A)** The GO enrichment analysis of the high-risk cluster (high-risk vs. low-risk) in the TCGA cohort; **(B)** Box plots show the StromalScore, ImmuneScore, and ESTIMATEScore in the TCGA and GEO cohorts; **(C)** Evaluation of the distribution of endothelial cells and fibroblasts in the two cohorts by MCPcounter and EPIC; **(D)** Activity scores of TGFb and WNT signaling pathways in the two cohorts. BP, biological process; CC, cellular component; MF, molecular function; ns, no significant; CAFs, cancer-associated fibroblasts; TGF-β, transforming growth factor-β. *p < 0.05; **p < 0.01; ***p < 0.001; ****, *p* < 0.0001.

To explore the heterogeneity between the Low-risk and High-risk clusters, the results of GO enrichment analysis revealed enrichment of extracellular matrix (ECM)-related processes (**Figure 5A**; **Supplementary Figure S4A**), such as extracellular matrix organization, extracellular structure organization, collagen-containing extracellular matrix, and extracellular matrix structural constituent. We then investigated whether the TME differed significantly between Low-risk and High-risk score clusters. Compared to the low-risk cluster, the ESTIMATE results showed a significant increase in the terms of StromalScore and ESTIMATEScore for the high-risk cohort, while there was no difference in ImmuneScore (**Figure 5B**). Specifically, MCPcounter and EPIC results showed that the High-risk cluster had a higher proportion of cancer-associated fibroblasts (CAFs) than the Low-risk cluster (**Figure 5C**). Further analysis showed that the transforming growth factor (TGF)-β and WNT pathways were significantly activated in patients with a High-risk score compared to those with a Low-risk score (**Figure 5D**; **Supplementary Figure S5A**). Most immune functions were not different between the two clusters (**Supplementary Figure S5B**). However, the levels of regulatory T cells, natural killer T cells, natural killer cells, and central memory CD4+ T cells were significantly higher in the High-risk cluster, whereas the levels of type 17 T helper cells were lower (**Supplementary Figure S4B**).

### Metabolic reprogramming and treatment response prediction

**Figure 6:**
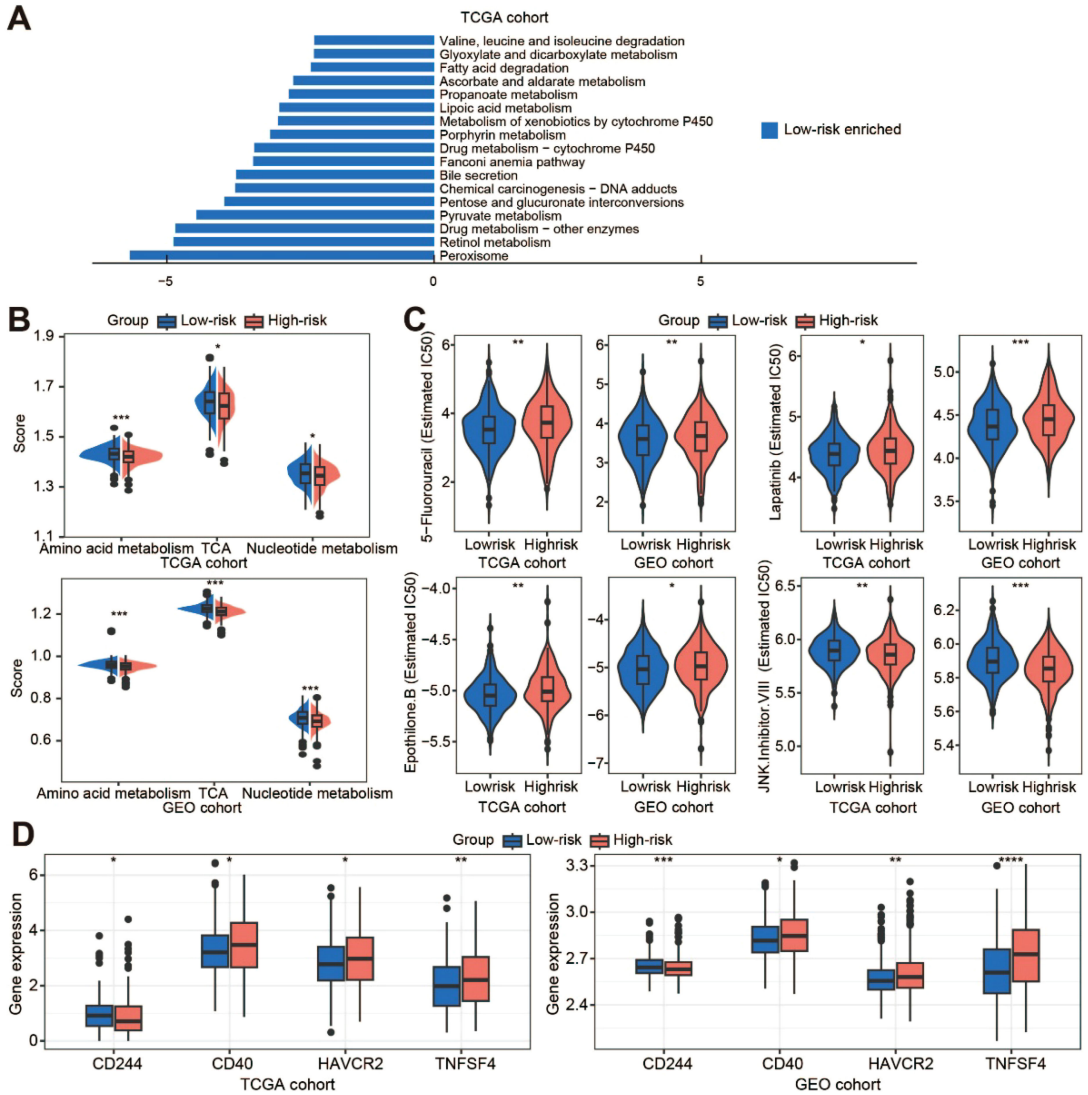
Metabolic reprogramming and treatment response prediction. **(A)** The KEGG enrichment analysis of the low-risk cluster in the TCGA cohort; **(B)** Comparison of metabolic levels in the low-risk and high-risk clusters of the TCGA and GEO cohorts; **(C)** Violin plots show the treatment response to chemotherapeutic drugs and inhibitors by TCGA and GEO samples; **(D)** The expression of immune checkpoint molecules in the two clusters of the TCGA and GEO datasets. TCA, tricarboxylic acid. *p < 0.05; **p < 0.01; ***p < 0.001; ****, *p* < 0.0001.

The results of KEGG analysis indicated that the Low-risk cluster was mainly enriched in metabolism‐related processes (**Figure 6A**; **Supplementary Figure S6A**). Metabolic reprogramming results showed that the Low-risk cluster had significantly higher metabolic levels of amino acid metabolism, tricarboxylic acid (TCA), and nucleotide metabolism (**Figure 6B**; **Supplementary Figure S6B**). The efficacy of some chemotherapeutic drugs and inhibitors was then compared between the two risk clusters. The IC_50_ values of 5-fluorouracil (5-FU), lapatinib, and epothilone B were lower in the Low-risk score cluster than in the High-risk score cluster, whereas JNK Inhibitor VIII was not (**Figure 6C**). The response of CRC patients to immunotherapy was also investigated. We performed somatic mutation analysis on the TCGA dataset and presented the OncoPrint plot of the TANs-related genes (**Supplementary Figure S6C**). There was no statistical difference in TMB between the two clusters (**Supplementary Figure S6D**). However, CD40, HAVCR2, and TNFSF4 were overexpressed and CD244 was low in the High-risk cluster (**Figure 6D**). In addition, the expression of CTLA-4/CD80/CD86 and CD274/PDCD1 was inconsistent between the TCGA and GEO cohorts (**Supplementary Figure S6E**).

## Discussion

Exploring TANs as a potential therapeutic target is still ongoing, as their function in cancer progression has not been fully elucidated. In this study, we investigated a novel type of TAN based on 18 genes in CRC, which showed significant prognostic value. Then, we constructed a comprehensive nomogram model by integrating the risk score with clinicopathological characteristics and demonstrated its significant clinical applicability. The High-risk score cluster was significantly associated with the stromal landscape and the level of CAFs. In depth, the High-risk score cluster showed a lower metabolic score and poorer responses to chemotherapeutic agents. To our knowledge, this study represents the frontier of a single-cell characterized prognostic signature combining TANs specific markers with clinicopathological features for prognostic stratification.

Neutrophils are short-lived cells with a rapid renewal rate, and they have diverse phenotypes depending on their environment. Due to the technical difficulties in handling neutrophils, most of the knowledge on neutrophil function in cancer has been derived from animal models. To date, the prognostic relevance of neutrophils has mainly been investigated using flow cytometry and immunohistochemistry. The specific markers, such as CD66b, have usually been used to isolate or distinguish neutrophils from peripheral blood or tumor tissue (15, 16). However, other unknown composition of neutrophils may not be fully explored by these methods. The recent development of scRNA-seq technology further is leading to a better understanding of the phenotypic heterogeneity and functional plasticity of neutrophils in cancer. During bacterial infection, eight neutrophil populations have been defined by distinct molecular signatures (17). Two neutrophil subtypes were also identified in esophageal carcinoma, showing greater enrichment in regulation of the inflammatory response, intercellular adhesion, and T cell activation (18). Four subpopulations of TANs have been identified in pancreatic ductal adenocarcinoma, with a terminally differentiated pro-tumor subpopulation correlating with poor prognosis (19). In non-small-cell lung cancer, four neutrophil subsets related to immune regulation and metabolism have been identified and a 6-gene prognostic model has been established (20). However, multiple markers of TANs for predicting CRC prognosis have not yet been identified. In this study, a novel single-cell characterized 18-gene signature was investigated to predict the survival of CRC patients.

The presence of TANs is significantly associated with poor prognosis in several cancers, including hepatocellular carcinoma (21), primary melanoma (22), localized renal cell carcinoma (23), and head and neck cancer (24). However, the prognostic significance of TANs in CRC remains controversial. Studies using non-specific staining methods, such as hematoxylin and eosin, have shown no correlation between neutrophil count and prognosis (25, 26). In contrast, the presence of CD66b^+^ intratumoral neutrophils has been associated with poor prognosis in CRC patients (27), whereas patients with higher density of myeloperoxidase-positive neutrophil density have been associated with better outcomes (28). These discrepancies may be due to the use of different markers, as well as the inherent heterogeneity and the plasticity of neutrophils in different inflammatory contexts. Consequently, polygenic profiles may offer more accurate prognostic predictions and greater clinical utility. In this study, we developed a risk score based on 18 TANs-related genes, which showed that patients with higher scores had shorter survival time. Meanwhile, our DCA results showed that the risk score provided a greater net benefit than existing clinical stage. In addition to prognosis, high densities of TANs have been correlated with more advanced disease in gliomas (29) and gastric cancer (30). Similarly, our risk score was significantly associated with T, N, and M stages. We constructed a nomogram model by integrating the risk score with clinicopathological features, which demonstrated a higher net benefit than the risk score alone.

The ECM is an important component of tumors and fulfils several essential functions such as modulating the microenvironment, offering mechanical stability, and serving as a reservoir for signaling molecules (31). Previous studies have demonstrated that neutrophils release neutrophil elastase (NE), cathepsin G (CG) and matrix metalloproteinases 9 (MMP9) to remodel the ECM, ultimately leading to tumor growth and metastasis (31). Our results showed that the risk score was related to the StromalScore and the function was enriched in ECM-related processes. MMP9 was mainly produced by the N2 TANs, and TGF-β modulates the N1 and N2 phenotypes of neutrophils. New strategies, including molecules targeting either TGF-β directly or its receptors, are currently in clinical trials (NCT02160106, NCT03620201 and NCT01058785). In addition to TGF-β, we have also found that the WNT pathway is associated with the TANs-based risk score, which may provide a novel potential therapeutic strategy for targeting TANs.

In clinical practice, ICIs have shown a significant effect in metastatic CRC patients with microsatellite instability-high/mismatch-repair-deficient, but limited response has been observed in CRC patients with microsatellite instability-low/mismatch-repair-proficient (32), possibly due to their different TME characteristics. As an important component of the TME, TANs may represent a significant substantial barrier to the success of the current immunotherapy. Previous studies have shown that TANs overexpress PD-L1 and suppress T-cell activation in gastric cancer (33). However, in our results, the risk score showed no direct association with the immune function, but exhibited significant correlations with CAFs. In hepatocellular carcinoma, CAFs mediated the immunosuppressive landscape and modulated the efficacy of combination therapy with PD-1 inhibitors and anti-angiogenic agents (14). This suggests that our TANs-based risk score may indirectly regulate immune function through stromal interactions. Intriguingly, increased levels of regulatory T cells, natural killer T cells, natural killer cells, and central memory CD4+ T cells, were higher in the high-risk patients, suggesting enhanced immunological activity and indicating “hot” tumor phenotypes. There are currently some clinical trials combining targeted neutrophils with immunotherapy, mainly focusing on PD-1/PD-L1 or CTLA-4 blockade, while most clinical trials have not yet shown significant results (34). We found that CD40, HAVCR2, and TNFSF4 were overexpressed and CD244 was low in the high-risk cluster. This may provide a novel combination approach for immunotherapy in CRC.

While mature neutrophils possess functional mitochondria, neutrophil metabolism remains incompletely characterized. Current evidence implicates multiple pathways, including glycolysis *via* the TCA cycle, pentose phosphate pathway *via* OXPHOS, glycogenolysis, glutaminolysis, and fatty acid β-oxidation, are thought to be used by neutrophils to meet the energetic, biosynthetic, and functional needs of neutrophils (35). In addition to TCA, our results showed that amino acid metabolism and nucleotide metabolism were enriched in the low-risk cluster. Metabolic alterations, a hallmark of cancer, enable tumor cells to adapt to their environment by modulating glucose, lipid, and amino acid metabolism, which promotes rapid growth and contributes to treatment resistance (36). Recent studies have shown that altered amino acid metabolism is tightly linked to tumor growth, metastasis, and therapeutic resistance by controlling the fate of various immune cells (37). Many of the aggressive behaviors of cancer cells, including chemotherapy resistance, are highly dependent on nucleotide metabolism (38). Similarly, our results showed that the IC_50_ values of 5-FU, lapatinib, and epothilone B were significantly higher in the high-risk cluster than in the low-risk cluster. As an essential component of systemic chemotherapy for CRC, the efficacy of 5-FU is critical in clinical practice (39). Therefore, our risk score could also contribute to the prediction of 5-FU responsiveness in CRC patients.

This study has certain limitations. Firstly, the limitations of scRNA-seq technology must be considered, such as its sensitivity to sample quality and the complexity of data analysis. There is also the potential for dropout events, whereby genes are not detected despite being expressed. Secondly, while we have shown that the risk score influences metabolic reprogramming and drug resistance, further studies using approaches such as bulk RNA sequencing, proteomics and metabolomics are required to clarify the mechanisms and reinforce our findings. Thirdly, our nomogram model based on TANs lacks verification from large-scale prospective studies.

In conclusion, we developed a risk score consisting of 18 TANs-related genes and demonstrated its potential for prognostic stratification. Mechanistically, the risk score exhibited significant correlations with ECM-related processes, CAFs infiltration, and metabolic reprogramming. Clinically, our findings demonstrate that the high-risk patients show increased susceptibility to chemotherapy resistance, highlighting its clinical relevance in predicting therapeutic outcomes.

## Data Availability

The original contributions presented in the study are included in the article/**Supplementary Material**. Further inquiries can be directed to the corresponding authors.
