# Supplementary material for: The prognostic model based on tumor-associated neutrophils contributes to the stromal landscape and influences metabolic reprogramming in colorectal cancer
Source: Front Immunol. 2025 Sep 2;16:1587947. doi: 10.3389/fimmu.2025.1587947 (PMC12436427; doi:10.3389/fimmu.2025.1587947)
Supplement: Supplementary file 1 [file DataSheet1.docx]

**Figure S1.**


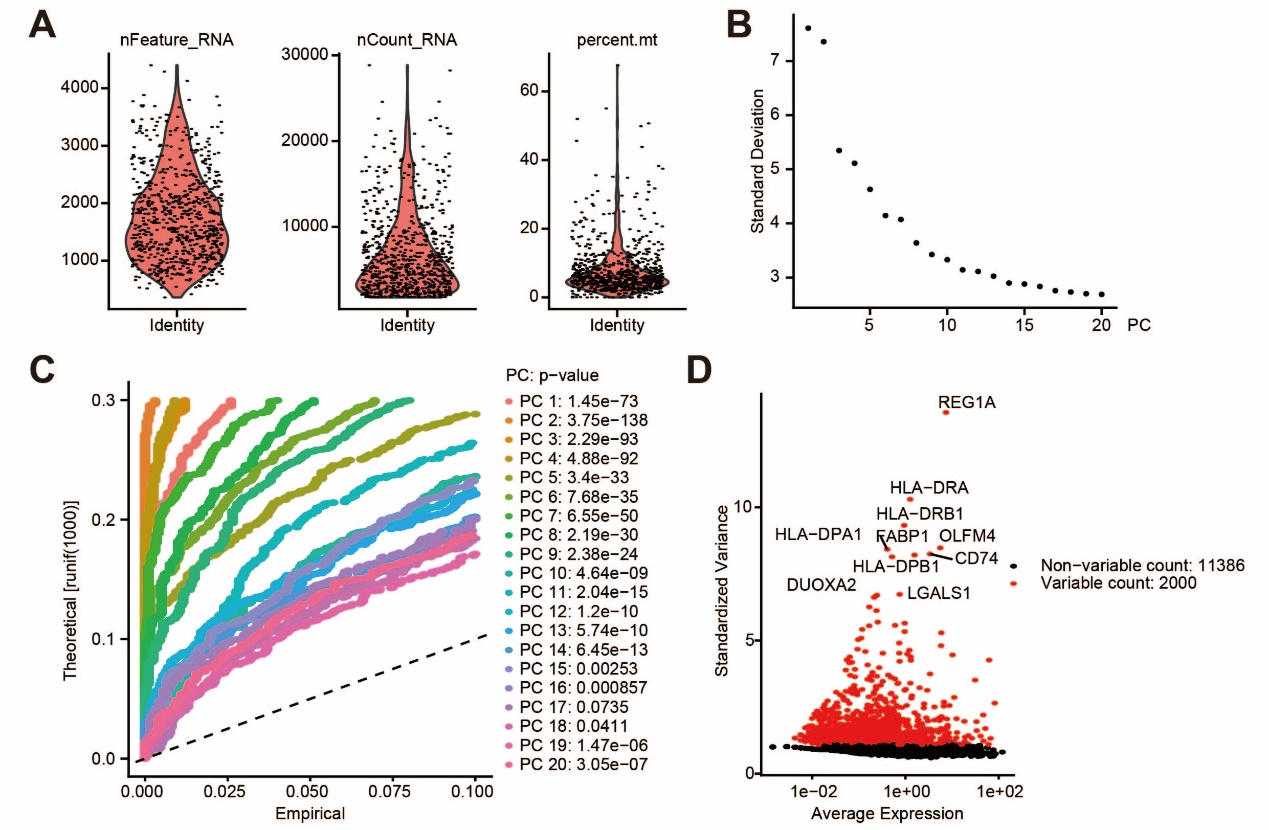


**Figure S1. scRNA-seq analysis.** (A) The sample quality control and the proportion of mitochondria; (B, C) The Elbow plot (B) and JackStraw plot (C) show the top 20 PCs; (D) The highly variable genes are showed and the top 10 genes are highlighted.

**Figure S2.**


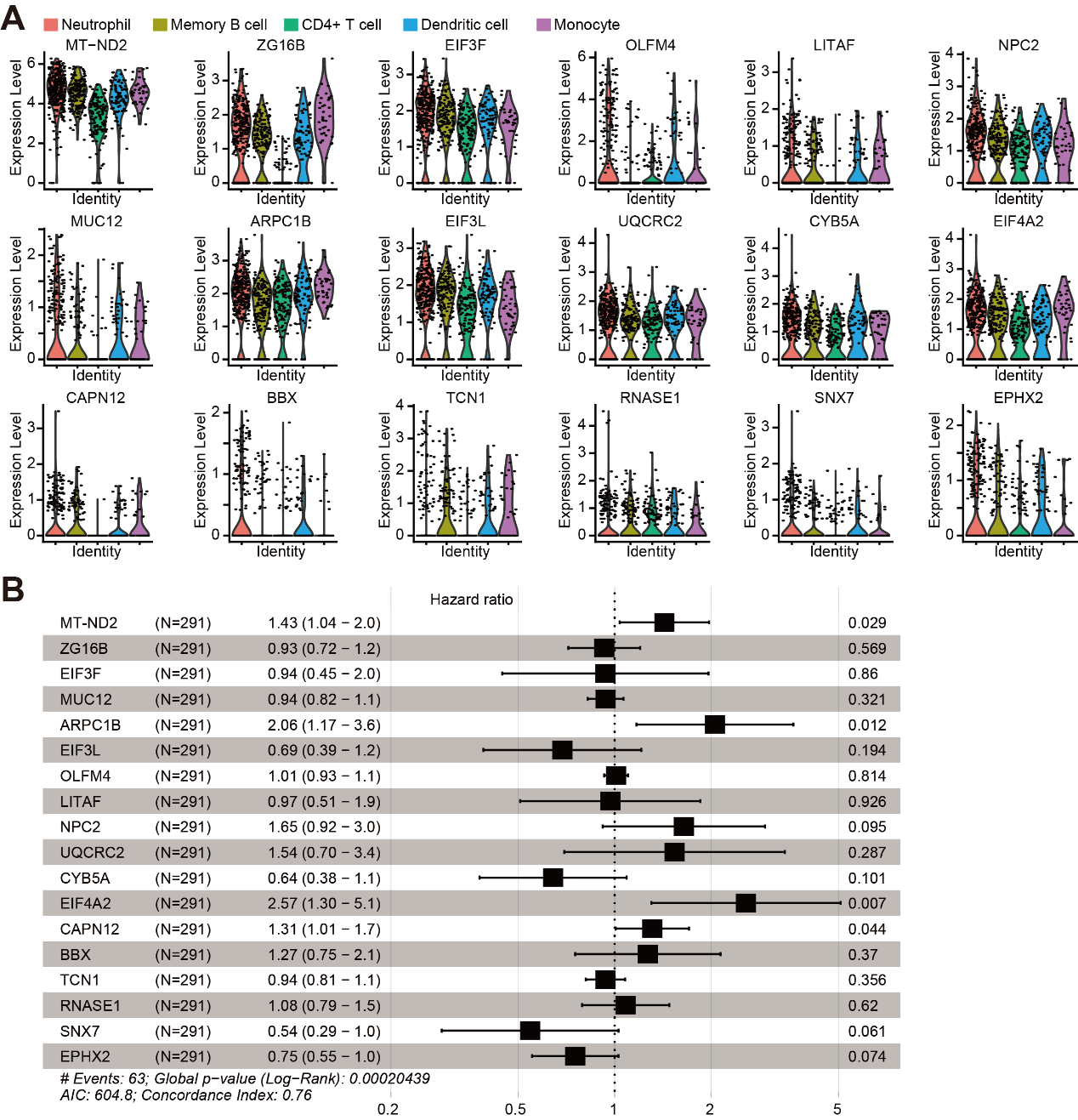


**Figure S2. Expression of the 18 TNAs survival-related genes and the risk score calculation.** (A) Expression of the 18 TNAs survival-related genes in the above five cell clusters; (B) Multivariate cox regression analysis of the 18 TNAs survival-related genes of the TCGA cohorts to obtain the prognostic model.

**Figure S3.**


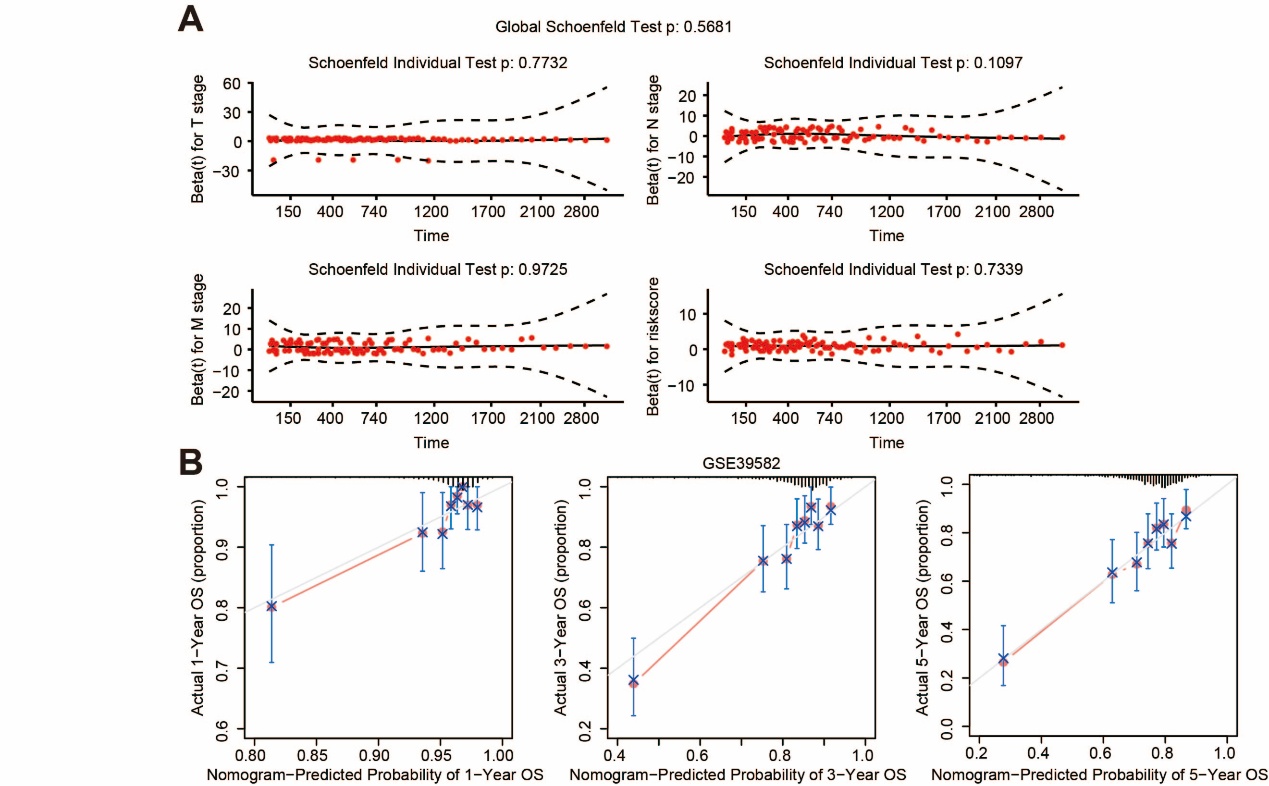


**Figure S3. Clinical application of the prognostic risk model.** (A) Test of the assumptions of the Cox PH model; (B) Decision curve analysis for the prognostic risk nomogram of the GSE39582 dataset.

**Figure S4.**


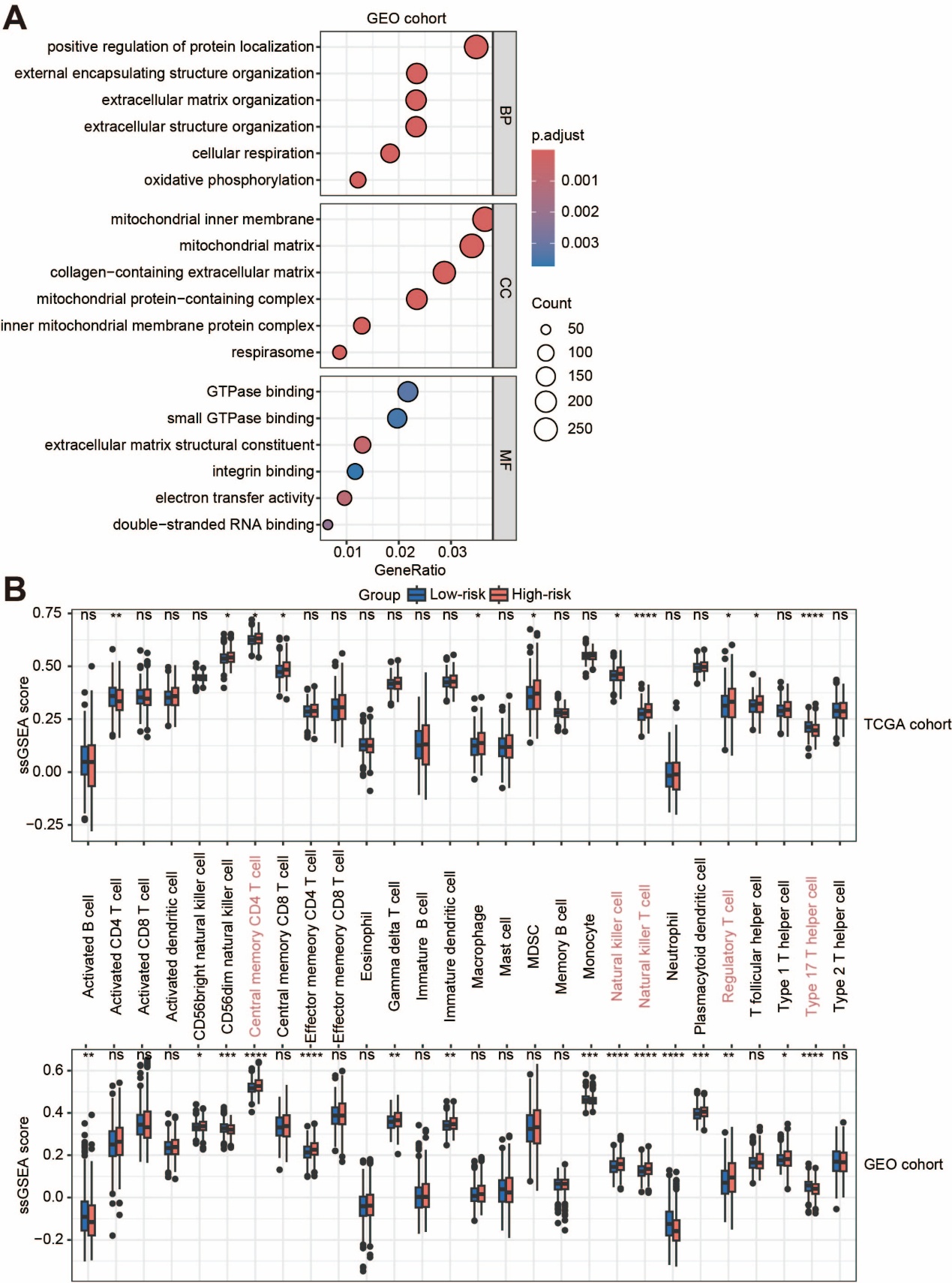


**Figure S4. Tumor microenvironment in the high-risk cluster.** (A) The GO enrichment analysis of the high-risk cluster (high-risk vs. low-risk) in the GEO cohort. (B) Comparison of 28 immune cells between the low-risk and high-risk clusters of the TCGA and GEO cohorts. Abbreviations: ns, no significant. *, *p* < 0.05; **, *p* < 0.01; ***, *p* < 0.001; ****, *p* < 0.0001.

**Figure S5.**


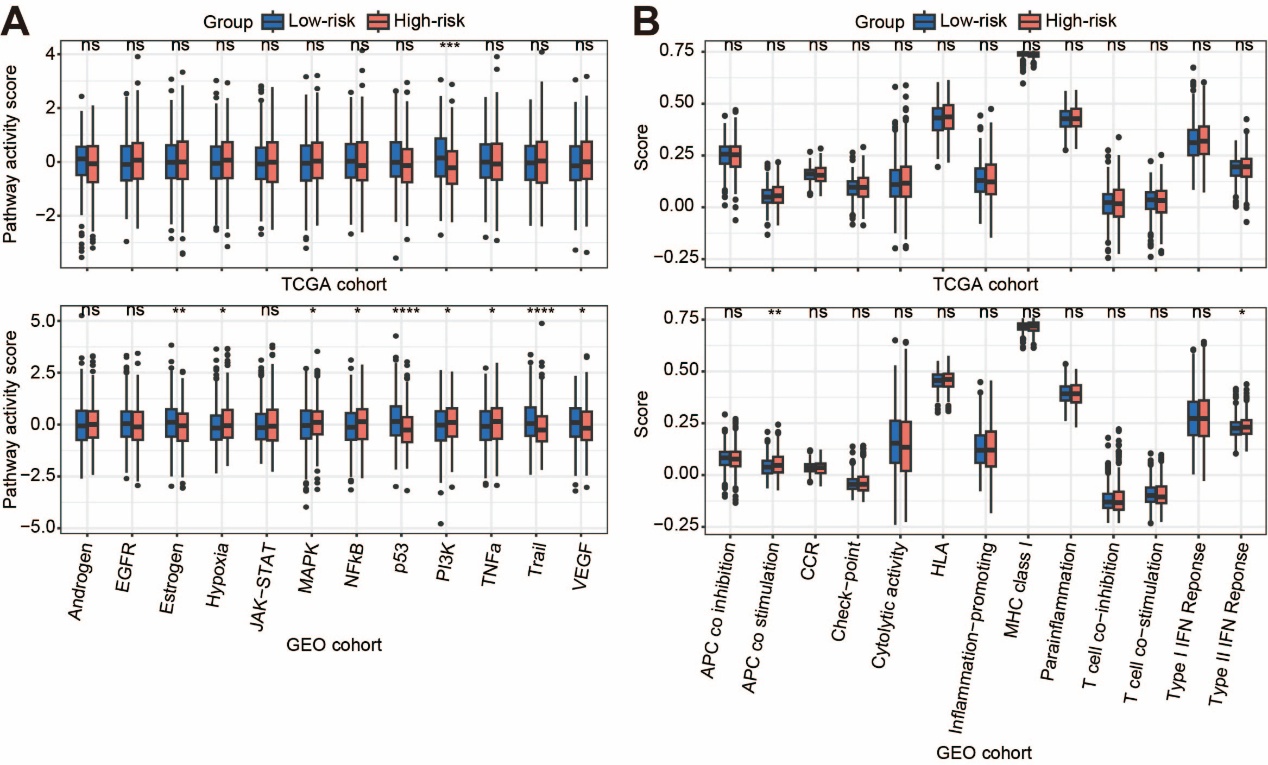


**Figure S5. Immune microenvironment and tumor-associated pathways.** (A) Activity scores of cancer‐associated signaling pathways in the two cohorts. (B) The distribution of immune function scores of the TCGA and GEO cohorts. Abbreviations: ns, no significant; *, *p* < 0.05; **, *p* < 0.01; ***, *p* < 0.001; ****, *p* < 0.0001.

**Figure S6.**


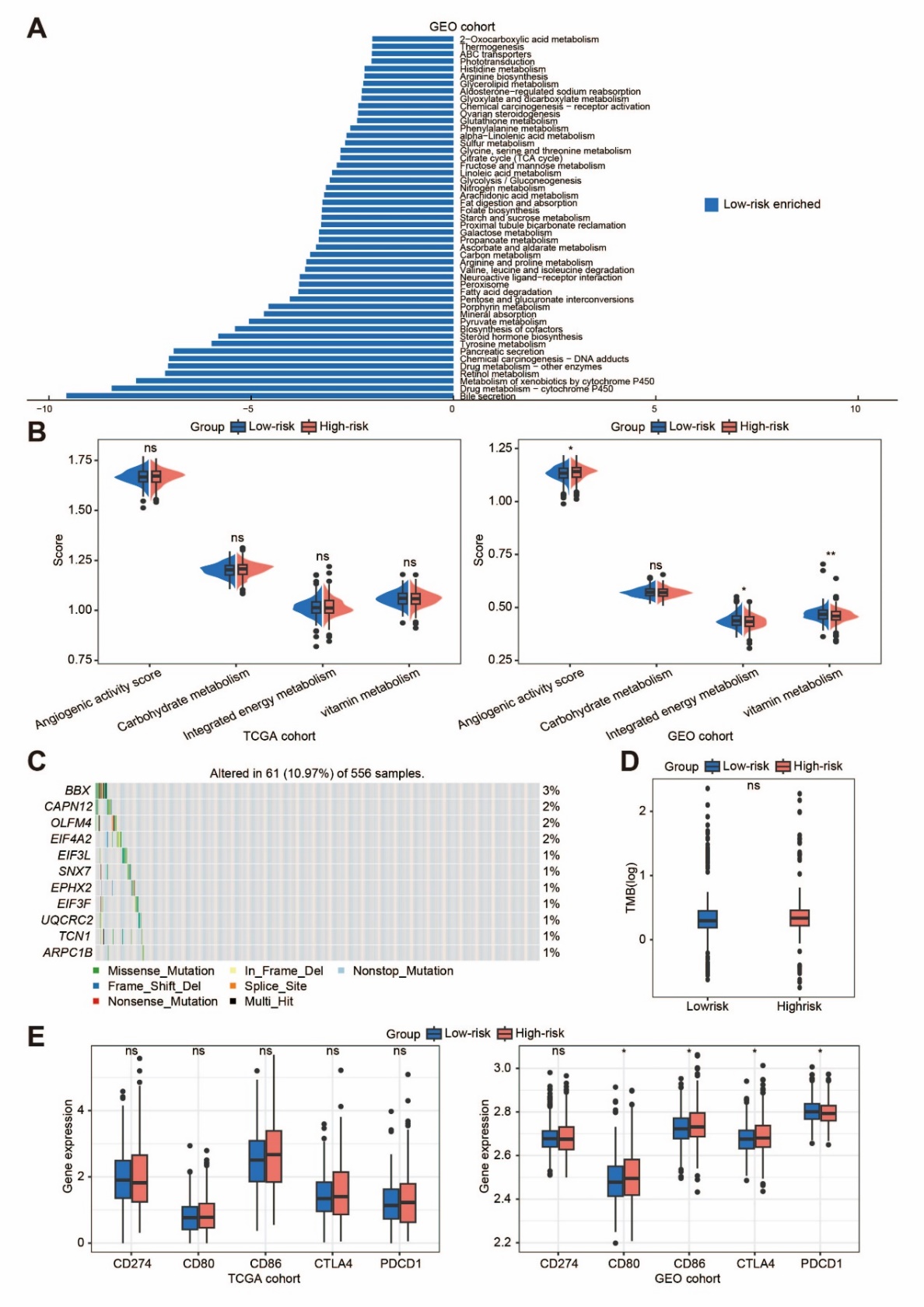


**Figure S6. Metabolic reprogramming and treatment response prediction.** (A) The KEGG enrichment analysis of the low-risk cluster in the GEO cohort; (B) Comparison of metabolic levels in the low-risk and high-risk clusters of the TCGA and GEO cohorts; (C) The oncoprint of the 18 TNAs survival-related genes of the TCGA cohort (genes without variations are not shown); (D) Boxplot showing TMB between the two clusters in the TCGA cohort; (E) The expression of immune checkpoint molecules in the two clusters of the TCGA and GEO datasets. Abbreviations: ns, no significant. *, *p* < 0.05.
