# Supplementary material for: The prognostic model based on tumor-associated neutrophils contributes to the stromal landscape and influences metabolic reprogramming in colorectal cancer
Source: Front Immunol. 2025 Sep 2;16:1587947. doi: 10.3389/fimmu.2025.1587947 (PMC12436427; doi:10.3389/fimmu.2025.1587947)
Supplement: Supplementary file 2 [file Table1.docx]

**Table S1.** TANs-related genes

| **Genes (133)** | | | | | |
| --- | --- | --- | --- | --- | --- |
| *LEFTY1* | *RPS4Y1* | *SELENBP1* | *LCN2* | *EEF1A1* | *CLDN2* |
| *ADH1C* | *GSTK1* | *MT-ND2* | *PRDX5* | *VSIG2* | *FAM3D* |
| *C15orf48* | *PABPC1* | *PIGR* | *GPX1* | *MT-CYB* | *CKB* |
| *RPL5* | *UGT2B17* | *MT-CO1* | *RPL9* | *MT-CO3* | *HMGCS2* |
| *SLPI* | *TMEM176B* | *ZG16B* | *MARCKSL1* | *GLTSCR2* | *EIF3F* |
| *RPL34* | *SCG5* | *CAT* | *MT-CO2* | *MUC12* | *FCGRT* |
| *ARHGDIB* | *MT-ND1* | *ARPC1B* | *PDZK1IP1* | *ATP1A1* | *EIF3L* |
| *GLUL* | *AKR1C3* | *CTSH* | *CCNI* | *ZFP36L2* | *OLFM4* |
| *FBP1* | *MT-ND4* | *IGFBP4* | *TXNIP* | *RPS3A* | *CMPK1* |
| *SOD3* | *TMEM219* | *TRAPPC6A* | *PLBD1* | *ECH1* | *ITM2C* |
| *ITM2B* | *ASCL2* | *IMPDH2* | *LITAF* | *ID1* | *COMMD6* |
| *ATP6V0E1* | *GRN* | *TMEM59* | *PMPCB* | *LYZ* | *RPL22* |
| *NPC2* | *TMEM176A* | *ARL6IP5* | *ST13* | *MMP7* | *LAPTM4A* |
| *PYCARD* | *UQCRC2* | *SH3YL1* | *MAGED2* | *CYB5A* | *EIF4A2* |
| *IFITM2* | *CXCL16* | *RSL1D1* | *CMBL* | *SYNGR2* | *CREG1* |
| *PPP1R1B* | *MT-ND3* | *SLC40A1* | *GSTM3* | *IGBP1* | *CAPN12* |
| *ABHD14B* | *PNRC1* | *GABARAPL2* | *RAB1A* | *GMPR* | *CHCHD10* |
| *BAX* | *APEX1* | *ATRAID* | *BBX* | *GPR160* | *BRK1* |
| *GBAS* | *QPCT* | *RPS27L* | *ARPC2* | *TP53TG1* | *CHMP3* |
| *ZFP36L1* | *DNPH1* | *PSAP* | *PRKAG2-AS1* | *TCN1* | *FXYD5* |
| *EEF1G* | *TMEM14C* | *RNASE1* | *DALRD3* | *PNKD* | *SNX7* |
| *HSD17B12* | *EPHX2* | *CTSB* | *STXBP6* | *FAM84A* | *CD44* |
| *TP53I3* |  |  |  |  |  |

Note: Genes in red indicate survival-related tumor-associated neutrophils genes selected from LASSO regression (18).
